# A New Prospective Solution to Meet the New Specifications Required on Agile Beam Antennas: ARMA Theory and Applications

**DOI:** 10.3390/s25113381

**Published:** 2025-05-28

**Authors:** Bernard Jecko, Pierre-Etienne Portalier, Mohamad Majed

**Affiliations:** Xlim (UMR CNRS 7252), University of Limoges, 87000 Limoges, France

**Keywords:** low-profile antennas, beam-agile antennas, beam forming, beam steering, wideband antennas, conformal antennas, multifunction antennas, high surface efficiency

## Abstract

**Highlights:**

**What are the main findings?**

- A more precise solution than phased arrays that pushes their main limits in all areas: surface efficiency, bandwidth, beam forming, beam steering, conformation, multifunctionality, focusing…

**What are the implications of the main findings?**

- New applications in terrestrial telecom (IoT, sensors, 5G, 6G…) and space ones (example: CubeSat), but also those in radar and electronic warfare (EW), require new performances for low-profile (LP) beam-agile antennas based on the points described in the previous paragraph.

**Abstract:**

The evolution of telecommunications and radars in the terrestrial and space domains is introducing new specifications for antennas that have difficulty meeting today’s phased arrays. Breakthrough technologies must be introduced to push back the limits not only in beam steering and beam forming, but also in frequency bandwidth, conformation, and multifunctionality. Indeed, the representation of radiating surfaces (Huygens) by arrays of point sources (a century ago!) is the poorest approximation of the rigorous solution, with well-known limitations. The proposed approach starts from the rigorous expression of the field radiated by any antenna obtained using the equivalence principle on any closed surface Sc surrounding the antenna. Important approximations are introduced to apply this rigorous result to the design of beam-agile multisource antennas that require sampling of the radiating Sc surface. The proposed approach samples the Sc surface by slicing it into small piecewise surfaces. For the fabrication of these small surfaces, structures called “pixels” deduced from the s have been designed. Many applications are proposed and compared with array solutions.

## 1. Introduction

Beam agility requires multisource antennas fed by a beam-forming network (BFN) [1]. On the antenna side, the most widely used current solution is the phased array technique, also known as AESA (agile electronically scanned array), which consists of designing elementary radiating sources, independents if possible, with phase centers representing the point sources of array theory (see Section 2.2).

These point sources are generally built by dipoles, monopoles, patches, slots, rings, etc. They are widely used in the design of axial fixed-gain antennas (non-agile beam) [1] and in beam agility, essentially for beam steering (Cf: Section 2.3) and beam forming (Cf: Section 2.4). In recent years, with the advent of meta-materials, more complex elementary sources [2] have been introduced, in particular with “parasitic elements” (the name is unjustified to the best of our knowledge,), to essentially widen array bandwidths. Under these conditions, it is no longer the array elements that radiate, but the multisource EBG antenna formed by the parasitic elements (FSS) fed by the array elements.

The solution proposed in this paper breaks away from the “ARRAYS” solution of sources, used worldwide and applied automatically when deducing the theory from the radiation of a point elementary source [3] (Cf: Section 2.2). The new approach starts from Maxwell’s equations with a better scan and therefore with an intrinsically better result.

## 2. Theory

### 2.1. Theory Introduction

Multisource planar antennas with beam agility are currently being used more and more for telecom (5G, sensors, space, IoT, etc.), radar, and EW applications.

The most widely used technique for performing these applications is called “phased arrays” or AESA. This approach introduces some limitations intrinsically linked to arrays: limited surface efficiency [4], narrow bandwidths, grating lobes, coupling effects, and high angle scanning limitation. This paper introduces a new approach, ARMA, to minimize these limitations [5].

**Figure 1:**
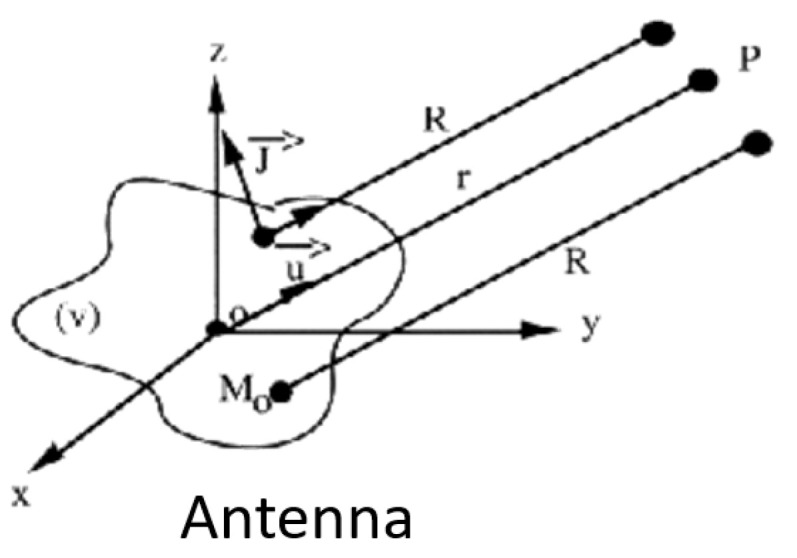
Radiating surface characterized by surface currents J(M_0_) used to calculate the far field $\vec{E}\left(P\right)$.

**Figure 2:**
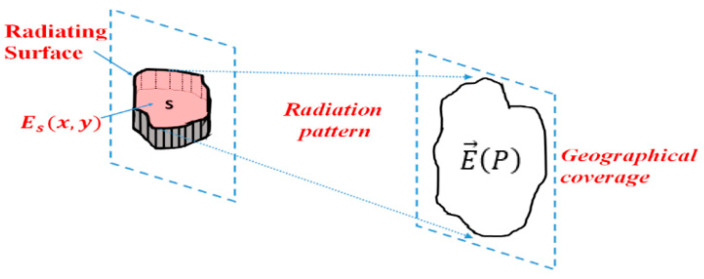
Example of an any close surface: the parallelepipedal one built with a metallic ground + metallic vertical walls + S (which is a PRS: partially reflecting surface).

Fortunately, the ideal solution to obtain the radiation pattern of any antenna is well known and is obtained by the following procedure:

- It is first derived from Maxwell’s equations, which give the far-field expression E(P) generated by surface currents J(M) located on a radiating surface S (Figure 1), as follows:
  (1) $$\vec{E}\left(P\right)=\frac{jk}{4\pi }\eta \psi \left(r\right)\underset{s}{\iint }\;\left[\left({\vec{J}}_{s}\wedge \vec{u}\right)\wedge \vec{u}\right]e^{j\vec{k}\vec{.OM_{0}}}ds$$
  (2) $$\psi \left(r\right)=\frac{e^{-jkr}}{r}$$
- Second, by applying the equivalent principle, it makes the field rigorously radiated to infinity E(P) by any antenna via the surface fields Es evaluated on any closed surface Sc (Figure 2) surrounding the antenna.
  (3) $$\vec{E}\left(P\right)=\frac{jk}{4\pi }\psi \left(r\right)\left(1+\cos\theta \right)\left(\cos\varphi {\vec{e}}_{\theta }-\sin\varphi {\vec{e}}_{\varphi }\right)\underset{Sc}{\iint }\;E_{s}\left(x,y\right)e^{jk\left(x\sin\theta \cos\varphi +y\sin\theta \sin\varphi \right)}ds$$

If you consider an Sc closed surface built under and with lateral metallic walls (cavity), almost all the energy is flowing through the upper PRS surface; then Sc ≈ S. Only the effect of currents flowing on the outer walls of the cavity is neglected. This approximation is valid for radiations around the antenna axis direction (normal to S), but more questionable for antennas that radiate away from the axis.

Furthermore, the integral on the S surface can be extended to the infinity because Es(x,y) = 0 outside the surface S. Consequently, the radiated field $\vec{E}\left(P\right)$ can be written as Equation (4).

(4) $$\vec{E}\left(P\right)=K\overset{\infty }{\iint }\;E_{s}\left(x,y\right)e^{jk\left(x\sin\theta \cos\varphi +y\sin\theta \sin\varphi \right)}ds$$

(5) $$\vec{E}\left(P\right)=K\;SFT\;\left(E_{s}\left(x,y\right)\right)$$

*SFT* is the Spatial Fourier Transform of $E_{s}\left(x,y\right);\;$then (Equation (6))

(6) $$\vec{E}\left(P\right)\approx Spatial\;Fourier\;Transform\;of\;E_{s}\left(x,y\right)$$

Remark: It is not exactly the SFT, due to (1 + cosθ) in the K factor.

### 2.2. Beam Agility

For a given law $E_{s}\left(x,y\right)$ on S, only one radiation pattern is obtained. For performing beam-steering or beam-forming, a lot of radiation patterns are needed; then the surface field expression $E_{s}\left(x,y\right)\;$must be sampled to be moved on S. The following two sampling techniques have been tested:

- The first one multiplies $E_{s}\left(x,y\right)$ in the integral (Equation (4)) by a Dirac comb [6] and leads to the well-known array technique [7] (Equation (7)). The radiated field appears as the sum of the radiations from point sources periodically distributed on the surface S. To physically represent these point sources, patches, slots, and dipoles are used, forming an array of elementary antennas.
  (7) $$\vec{E}\left(P\right)=K\underset{i}{\sum }\underset{j}{\sum }E_{s}\left(x_{i},y_{j}\right)e^{jk\left(x_{i}\sin\theta \cos\varphi +y_{j}\sin\theta \sin\varphi \right)}$$
- The second one quantifies the field by multiplying $E_{s}\left(x_{i},y_{j}\right)$ by the Π(x,y) function [6] (Equation (8)), the sum of rectangular functions on x and y (Equation (11)); this is the ARMA technique [5].
  (8) $$\Pi \left(x,y\right)=\underset{i}{\sum }\underset{j}{\sum }\pi \left(x-x_{i}\right)\pi \left(y-y_{j}\right)$$
  (9) $$E\left(P\right)=K\underset{i}{\sum }\underset{j}{\sum }\iint_{S}\;E_{i,j}\left(x,y\right)e^{jk\left(x\sin\theta \cos\varphi +y\sin\theta \sin\varphi \right)}\pi \left(x-x_{i}\right)\pi \left(y-y_{j}\right)dydx$$
  (10) $$E_{i,j}\left(x,y\right)=A_{i,j}e_{i,j}\left(x,y\right),with\;e_{i,j}\left(x,y\right)=1\;on\;s_{i,j}\;and\;zero\;outside$$
  (11) $$E\left(P\right)=K\underset{i}{\sum }\underset{j}{\sum }A_{i,j}\iint_{S}\;e_{i,j}\left(x,y\right)e^{jk\left(x\sin\theta \cos\varphi +y\sin\theta \sin\varphi \right)}dydx$$

**Figure 3:**
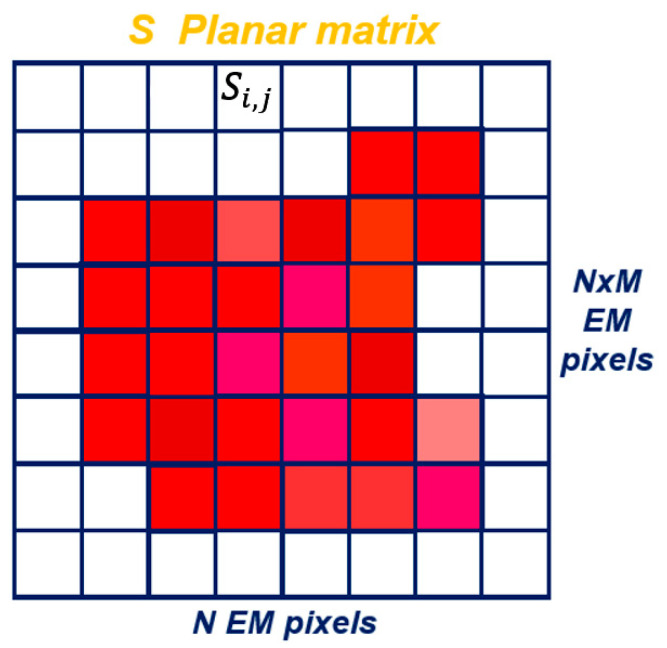
Example of an S Planar Matrix divided into small surface elements $s_{i,j}$ each characterized by a weighting coefficient $A_{i,j}$ (color). The surface field must be constant on each $s_{i,j}$ element.

Considering the special case of a planar surface S (rectangular for example), this surface is divided into small surface elements $s_{i,j}$, building a matrix with N × M elements. Each $s_{i,j}\;$is fed by a weighting value $A_{i,j}\;$corresponding to the surface field expected on the element $s_{i,j}$ [6] (Figure 3). The obtained sampled radiating surface field introduced in Equation (11) gives a radiation pattern, which can be moved (agility) only by changing the $A_{i,j}$ coefficients.

### 2.3. Manufacturing

**Figure 4:**
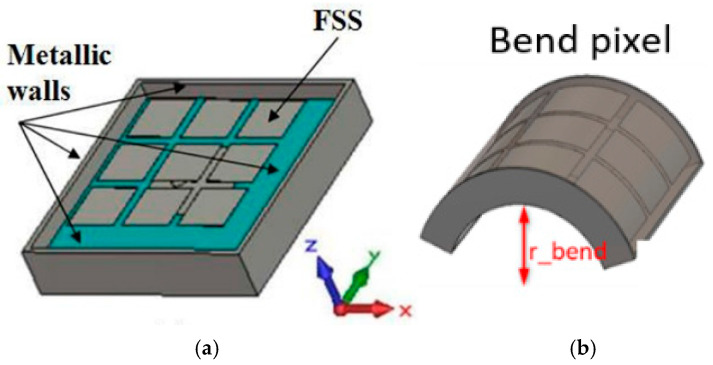
Example of a pixel: (**a**) planar square shaped; (**b**) bend square shaped.

To design the $s_{i,j}$ surface (characterized by an $E_{i,j}\left(x,y\right)$ field uniform in amplitude and phase), a physical structure capable of exhibiting this uniform field must be found (Cf: Section 2.5): the pixel. The pixel is a small metal cavity whose upper face is made by a PRS, which can be an FSS (Figure 4), a metasurface, or a dielectric slab.

**Figure 5:**
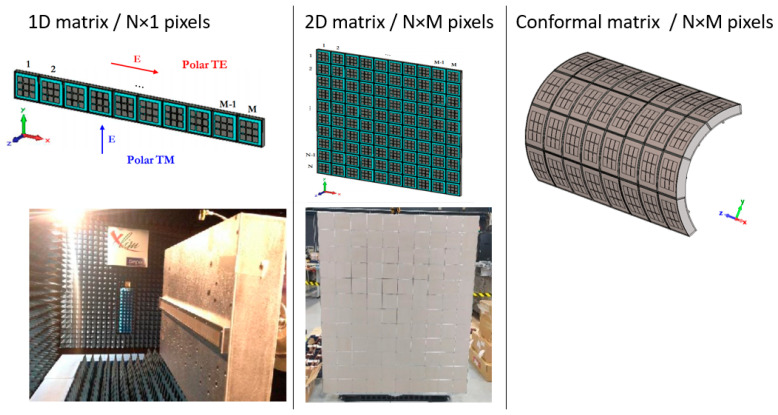
Examples of ARMA antennas obtained with square-shaped cuboid (parallelepipedal) pixels (planar or bent pixels).

**Figure 6:**
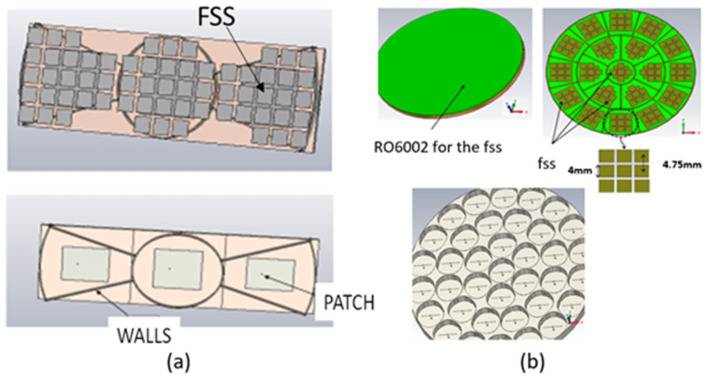
(**a**) Some pixels of any shape: circular and trapezoidal ones with and without the PRS (FSS); (**b**) circular matrix applications, RFID example to switch between different directions (upper view). Building a circular symmetry antenna working in linear polarization shown without the upper PRS (lower view).

Many pixels are joined together to build a radiating matrix of any shape. Some examples are given in Figure 5 with square-shaped pixels, as follows:

Additionally, pixels of any surface shape can also be designed. For example, pixels with a circular, elliptic, or trapezoidal S surface (Figure 6a) build a lot of ARMA for RFID applications.

### 2.4. Polarization

**Figure 7:**
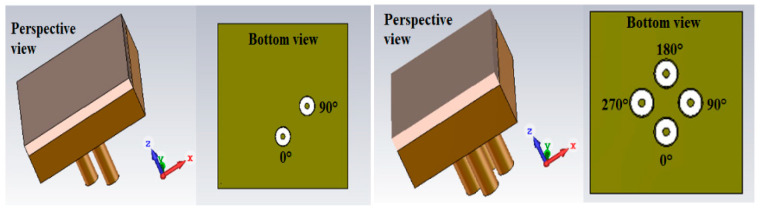
Two and four ports used to feed a patch probe inside the pixel.

The polarization of the pixel follows the polarization of the probe. Then all the polarizations can be obtained for the pixel (Section 2.5). They are usually introduced by one or many appropriate ports inside each pixel. An example of a pixel in circular polarization is given Figure 7.

### 2.5. Pixel Design Theory

As said before, the pixel (i,j) must be able to exhibit on its upper surface $s_{i,j}$ a uniform field in modulus and phase and zero outside. Its design is deduced from EBG resonator antennas [8] in the “Low profile” version [9,10,11].

**Figure 8:**
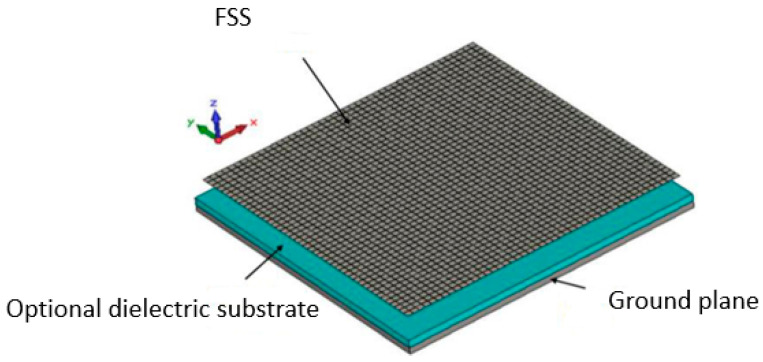
Low-profile EBG antenna built with an FSS, an optional dielectric substrate on which a patch probe is printed.

The EBG resonator antenna, in its simplest form, is an antenna constituted by a ground plane (horizontal) and above a parallel PRS (Figure 8) fed by a probe located in the middle of the structure.

**Figure 9:**
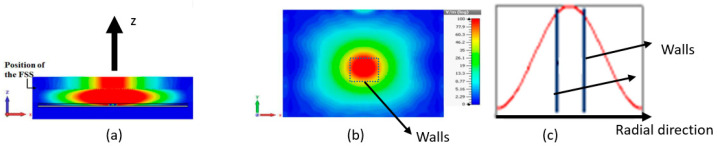
Field amplitude cartography (**a**) in a vertical plane and (**b**) on the roof of the structure and (**c**) field evolution in the radial direction of the EBG antenna where vertical walls are introduced to limit the approximately constant $E_{s}$ field.

The E field cartography inside such a structure shows that all the energy flows into the z direction (Figure 9a); the E field is vanishing in the r direction (Figure 9b), exhibiting an area where the field amplitude is approximately constant (circular spot). More precisely, the field evolution as a function of the radial direction (Figure 9c) shows that the field is approximately constant near the z axis.

**Figure 10:**
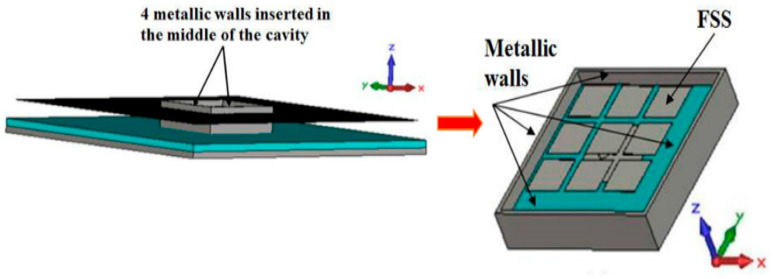
Square-shaped pixel obtained by limiting the structure with the four walls (the same result shown in Figure 4).

In these conditions, vertical metallic walls can be introduced to build a small cavity exhibiting a constant field at its upper surface. That is the pixel (Figure 10).

**Figure 11:**
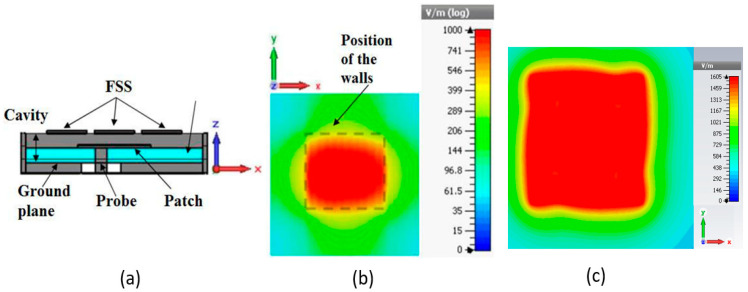
(**a**) Square-shaped pixel fed by a patch probe; (**b**) surface field cartography (modulus) in linear polarization. (**c**) Same kind of result with a circularly polarized probe.

Finally, the pixel is fed by a probe, for example, a patch, as shown in Figure 11. Then the pixel exhibits at the upper surface a constant field. This behavior remains almost identical when the pixel is inserted into a matrix due to the transverse evanescence of the modes.

**Figure 12:**
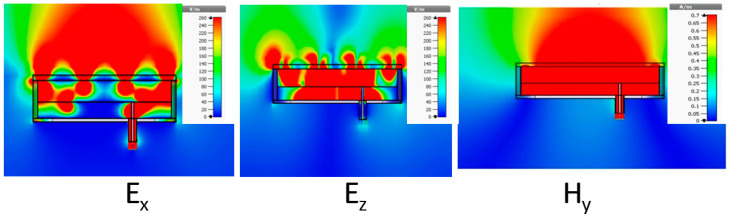
Mapping of non-zero fields in the pixel at its central working frequency for an ox linear feeding patch.

The mapping of EM fields within the pixel illustrates well the behavior of this structure on the TM_1_ EBG mode. Cartographies are taken at the band center frequency but are similar for all the frequencies of the working band. For an “ox” feeding source, the components E_y_, H_x_, and H_z_ are zeros; Figure 12 shows the other components: E_x_, E_z_, and H_y_.

### 2.6. ARMA Construction

**Figure 13:**
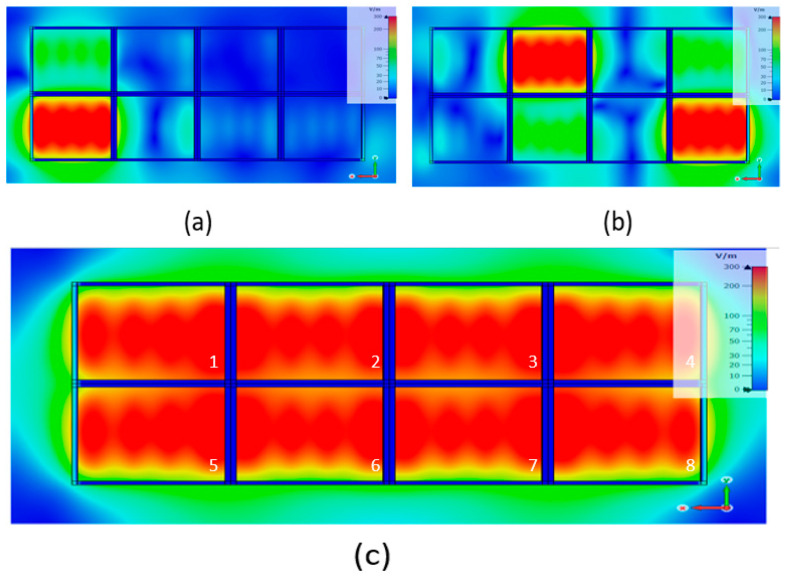
Example of the surface field amplitudes obtained in a 4 × 2 matrix uniformly fed ($A_{i,j}$ = 1): (**a**) only the 5th port is fed, (**b**) with the 2 and 8 ones, (**c**) with all the ports.

Pixels are joined together to build the matrix, and then, a large radiating surface S appears, built with the $s_{i,j\;}$elements exhibited by each pixel. The radiating surface is constructed (Figure 13) by adding the contributions (surface field on $s_{i,j}$) of each pixel inside the matrix and not radiating alone, as is the case in the array. In Figure 13c, the radiating surface built with the $E_{s}\left(x,y\right)$ fields is almost a uniform radiating surface (aperture), which gives an axial gain equal to 4πS/λ^2^. It is therefore obvious that the surface efficiency ἠ (defined by the ARMA axial directivity divided by the previous gain) of ARMA will be close to 100%. This coefficient does not excess ≈ 80% for an array with the same surface.

## 3. Some Applications and Comparisons with Phased Arrays

### 3.1. Large Bandwidth

#### 3.1.1. Principle

**Figure 14:**
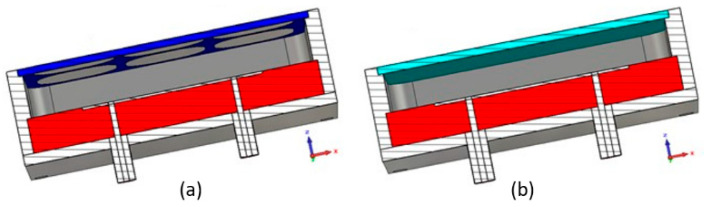
(**a**) L-band pixel whose PRS consists of an FSS (frequency selective surface) printed on a substrate, ξ = 3.55, tanδ = 0.001. (**b**) Pixel in the Ka band whose PRS is a dielectric layer made of Zirconia ξ = 23, tanδ = 0.002.

The bandwidth of ARMA is deduced from that of its constituent pixels. Likewise, the bandwidth of each pixel is directly related to that of the native EBG antenna (Section 2.5). In its low profile version, the EBG antenna has a negative reflection coefficient phase of its PRS, which leads to a resonator with a low-quality coefficient. Therefore, it presents a large frequency bandwidth that is obviously found in the pixel. For example, two pixels built with different PRSs and working in different frequency bands are shown in Figure 14. In this picture, they are excited by a patch probe fed with two ports with a 180° phase offset to maintain symmetrical behavior.

**Figure 15:**
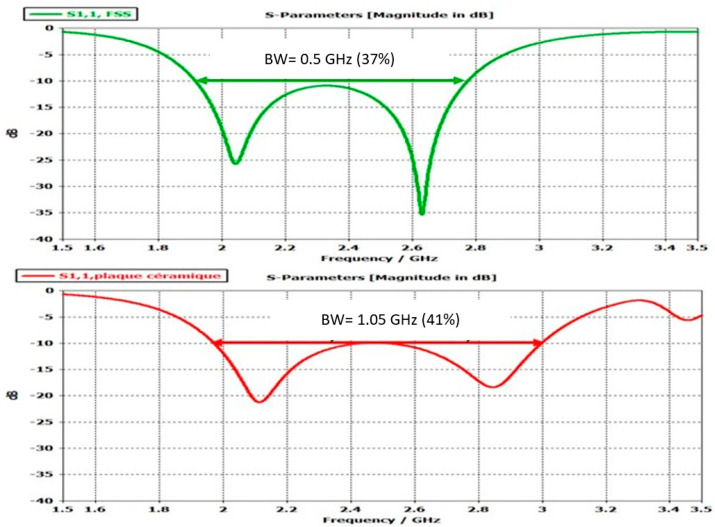
Wide band reflection coefficient of the pixels from Figure 14.

In addition, a pixel’s bandwidth is very wide ≈40% (Figure 15). Such bandwidths are inaccessible by phased arrays based on patches, or slots Their bands are limited to ≈15%. To widen the band, some authors [2] introduce in the array patches called “parasitic” ones or more complicate elements built with meta-materials. In this case, it is no longer the array patches that radiate but the EBG multisource antenna [12] formed by the FSS composed of the parasitic patches and fed by the array patches.

**Figure 16:**
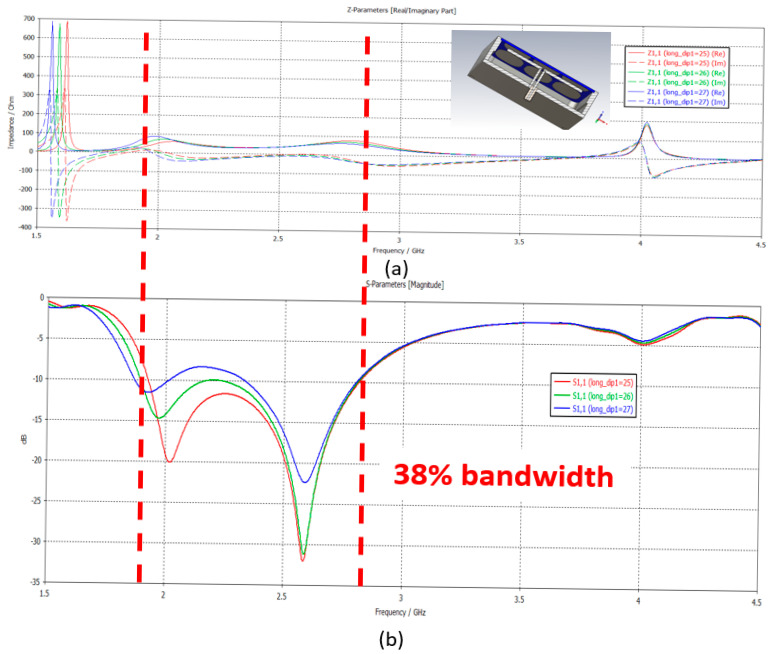
(**a**) Real and imaginary parts of the pixel impedance as a function of the frequency and (**b**) S11(f) evolution.

To isolate individual elements, some authors also introduce vertical walls and fall back on ARMA pixels. This assertion is illustrated in Figure 16 by studying the evolution of the reflection coefficient versus the frequency of a pixel fed by a dipole (the result will be the same when it is fed by a patch). When the length of the dipole is changed: the resonance position of the dipole changes following its length (Figure 16a), whereas the matching band of the pixel (corresponding to the TM_1_ mode of the EBG antenna) remains unchanged whatever the feed is (Figure 16b).

**Figure 17:**
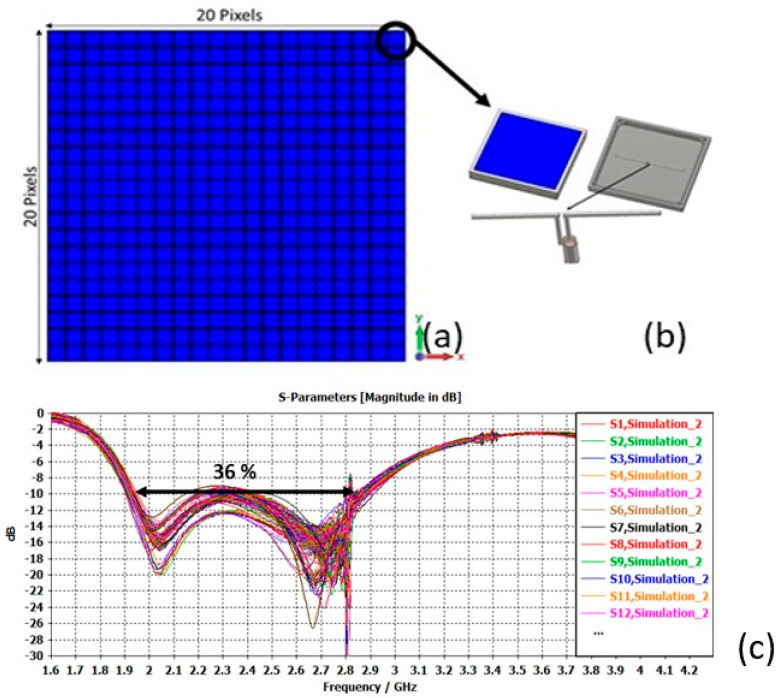
(**a**) ARMA antenna, (**b**) the pixel, (**c**) evolution of the reflection coefficient for each pixel feeding the matrix.

Finally, if we consider a high-gain axial-radiation matrix with a large number of pixels, each pixel remains matched over the whole band (Figure 17).

**Figure 18:**
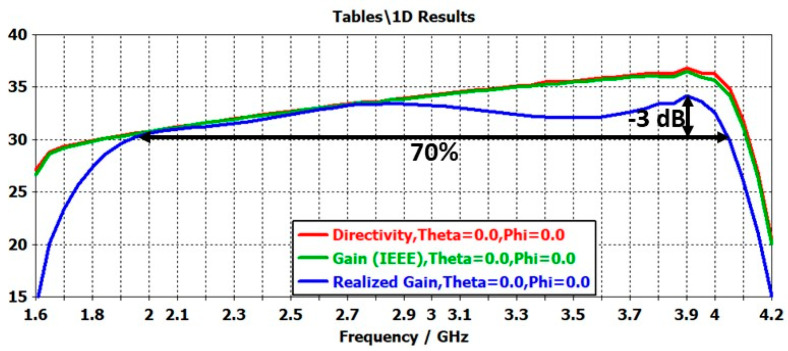
ARMA 20 × 20 antenna: directivity, gain, and realized gain as a function of frequency.

Under these conditions, the ARMA matrix offers excellent axial directivity, intrinsic gain (IEEE), and realized gain characteristics over a very wide frequency range. It is leading to a surface efficiency very much greater than that of an array (Figure 18).

Note that the matching of the pixels must be preserved when the beam is steered to high-angle directions (Section 2.3). It is not the case of the previous ARMA 20 × 20, designed for on-axis pointing.

#### 3.1.2. Hole Generation in the Large Pixel Band

**Figure 19:**
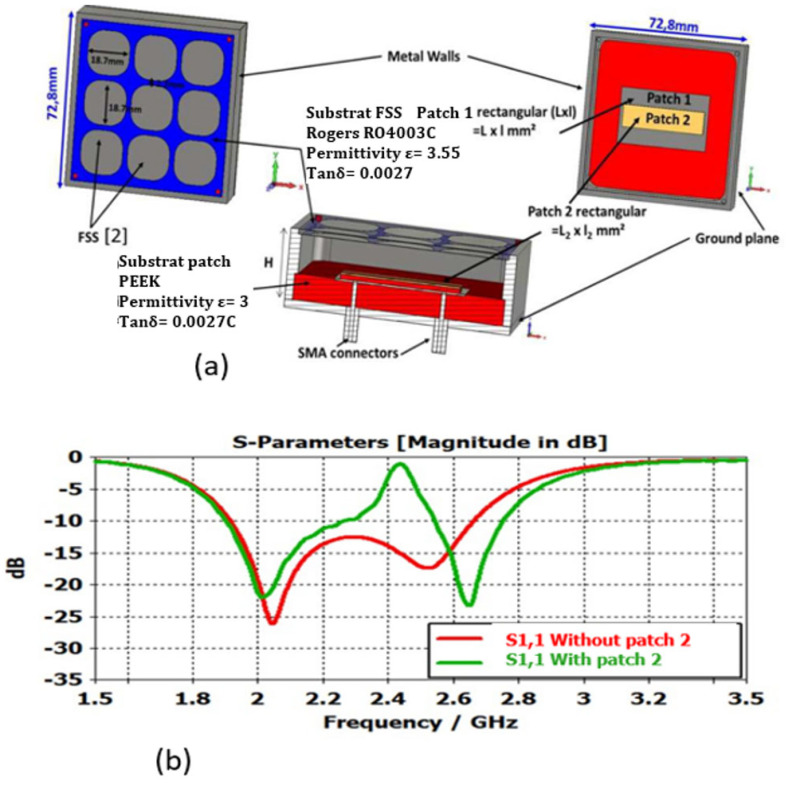
(**a**) Pixel with a second patch (the colored materials are dielectrics substrates); (**b**) reflection coefficient for a pixel with and without the second feeding patch.

What is more, it is possible to divide this very wide band into sub-bands by creating holes in the frequency band. The technique consists in introducing into each ARMA pixel a second passive patch (or dipole) excitation just above the first one and parallel to it. The second patch exhibits a surface field (or current) in the opposite direction with that of the first patch. A very weak field appears in each pixel for frequencies corresponding to a resonance of the second patch linked to its length L2 (Figure 19a). The evolution of the reflection coefficient as a function of frequency for each pixel shows a region characterized by short-circuit behavior, as shown in Figure 19b.

**Figure 20:**
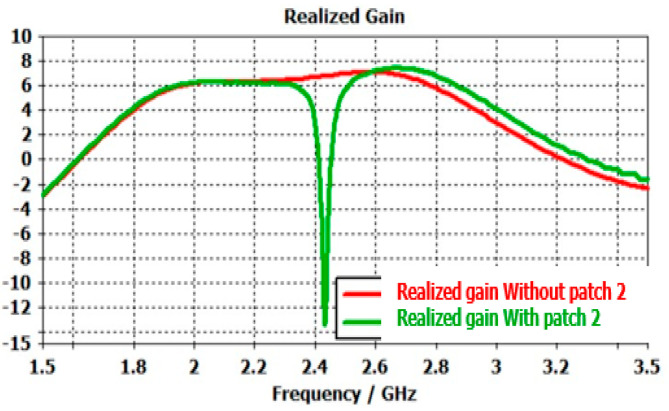
Frequency gain evolution of an ARMA realized with only one pixel.

Any ARMA built with such pixels exhibits a gain evolution as a function of the frequency with two or more frequency bands, as shown in Figure 20 for an ARMA reduced to one pixel to simplify.

This technique is also very effective for protecting narrow-band communication antennas from the radiation of a broadband ARMA located on the same platform and working in the same frequency range.

### 3.2. Beam Forming

**Figure 21:**
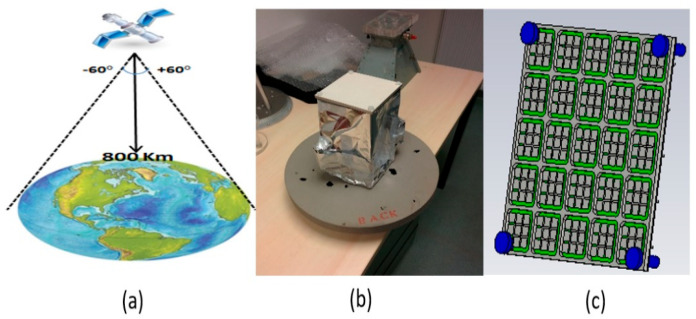
(**a**) Isoflux coverage, (**b**) CubeSat with the antenna, and (**c**) 5 × 5 pixels ARMA.

ARMA beam forming applications are very wide-ranging, so an example is chosen in the space domain of isoflux terrestrial coverage from a low-elevation (LEO) satellite (CubeSat) working in X-band with a circular polarization (Figure 21). A mask defining the radiation pattern to be obtained shows great difficulty in generating gain maxima around 60° and a hole in the antenna axis. Indeed, the dimensions of the antenna support to be integrated on the CubeSat only allow multisource antennas with five ports in one direction, i.e., 5 × 5 ports for the whole antenna [13].

**Figure 22:**
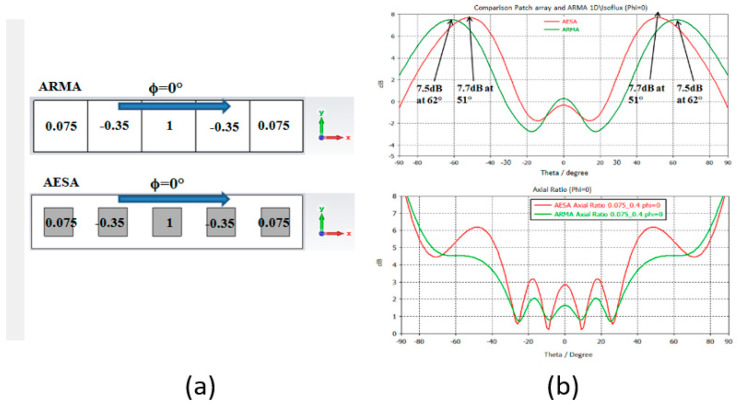
ARMA–AESA comparison in two 1D antennas with 5 elements supplied by the same isoflux law: (**a**) radiation pattern and (**b**) axial ratio.

To compare with a 5 × 5 patch antenna array, an approach was carried out on 1D antennas corresponding to one row of the 2D antennas (Figure 22a) fed by the same Ai,j law.

**Figure 23:**
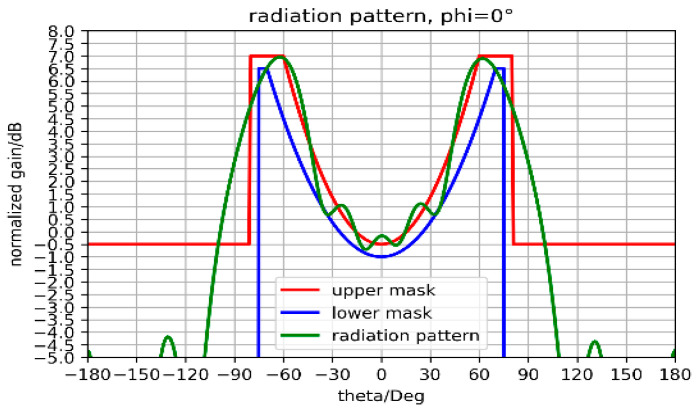
Isoflux coverage between upper and lower mask obtained by a similar matrix using an optimization algorithm.

A large difference in the position of the maxima of the radiated field obtained with ARMA and AESA (10°) is due to the better sampling (Section 2.2) with ARMA, as illustrated in Figure 22b, which also shows that the evolution of the axial ratio as a function of θ is better with ARMA. Recently, the same results have been obtained using an optimization algorithm, as shown in Figure 23.

**Figure 24:**
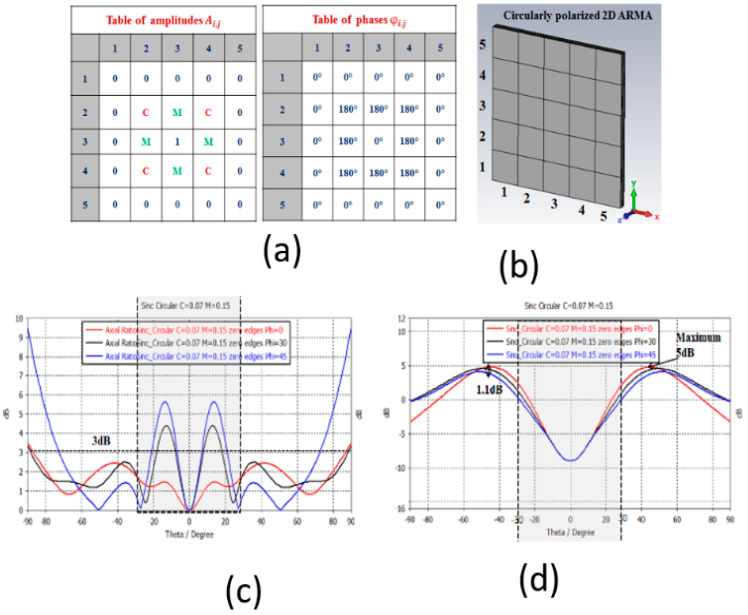
(**a**) Isoflux ARMA 5 × 5 weighting laws, (**b**) ARMA 5 × 5 structure, (**c**) axial ratio for different φ direction, and (**d**) radiation pattern for different φ direction.

This behavior is reflected in the 2D antenna shown in Figure 24. The rotational symmetry is difficult to obtain with a small square antenna by changing only the Ai,j coefficients.

To solve the symmetry problem on the radiation pattern, it is better with ARMA to consider a circular matrix (Figure 6).

### 3.3. Beam Steering

Pointing a beam in directions far from the axial direction of a planar agile beam antenna with few elements in each direction (cost, weight) is a challenge for many applications, while keeping the gain maximum within 3 dB of that obtained on axis for all the frequencies of the band.

The main point to study concerns linear 1D antennas, as the study in 2D will depend on the shape of the antenna, introducing other limitations. In this 1D case, the pointing directions of the beams obtained with ARMA and AESA are compared. It was shown, in the Introduction (Section 2.1), that the radiated field is the Fourier transform of the surface field located just above the 1D antenna. Therefore, to shift a beam, the property of modulating [6] a function by an imaginary exponential that amounts to translating its FT can be applied [7], provided that the number of ports is sufficient to limit edge effects.

**Figure 25:**
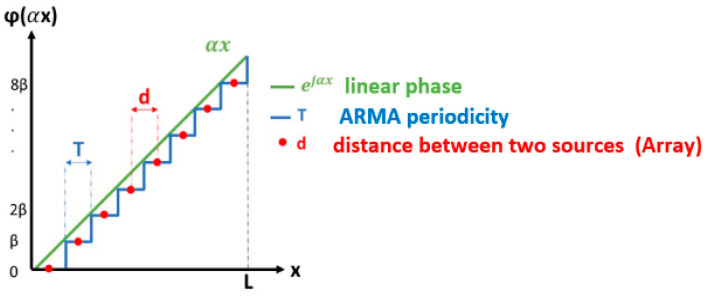
Linear phase shift approximation with ARMA and AESA.

The comparison between ARMA and AESA 1D antennas clearly illustrates the advantage of better sampling for ARMA, which better approximates the ideal phase law (Figure 25).

**Figure 26:**
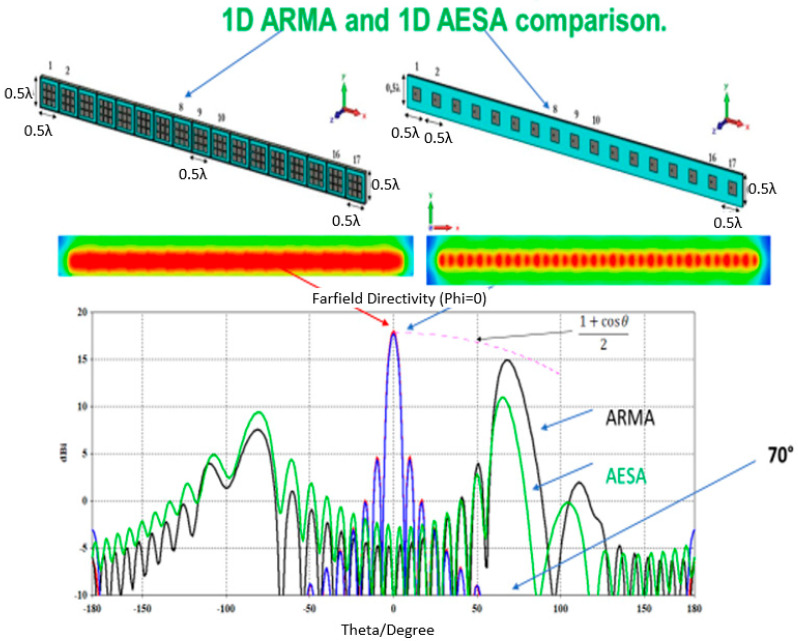
Comparison of two 1D antennas, ARMA and AESA, pointing in the axial direction and in the 70° direction.

Let us consider two 1D ARMA and AESA antennas with the same surface area, the same periodicity (chosen to see the periodicity lobes), and therefore the same number of elements and fed with the same steering law. The radiation patterns are compared in Figure 26.

- First, in the axial direction, the surface field amplitude of Es on ARMA is more uniform than this one on the array, but this difference has very little influence on the gain evolution as a function of θ.
- Second, in the 70° direction, the results are very different: the maximum gain with ARMA roughly follows the 1 + cos(θ)/2 law of Equation (1), while the maximum gain with AESA drops by around −4 dB.

### 3.4. Conformal Antenna

**Figure 27:**
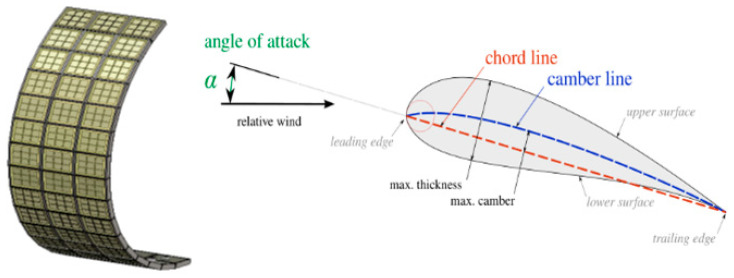
16 × 3 ARMA embedded on the leading of an aircraft.

Planar antennas can be designed for installation on non-planar surfaces. For example, embedding a 16 × 3 directive planar ARMA on the leading edge of an aircraft wing completely destroys directivity. The conformal ARMA antenna is introduced (Figure 27).

**Figure 28:**
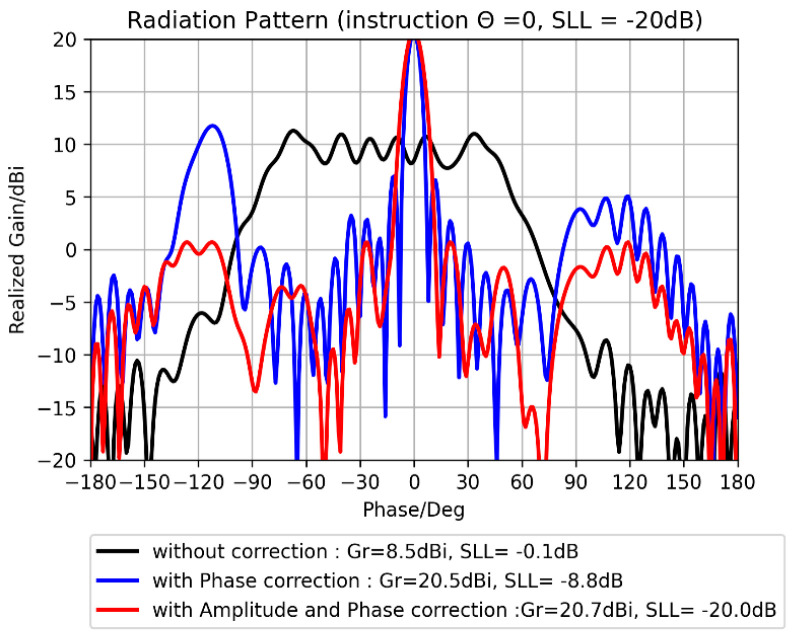
ARMA radiation pattern corrected by the optimization technique.

To find the max gain in a desired direction, an optimization method has been developed to calculate the suitable coefficients Ai,j. If the side lobe level is too high, a constraint on them can be added (Figure 28).

A comparison with an array has already been demonstrated before for beam steering and beam forming on planar antennas, but in addition, a conformal antenna on a non-planar surface leads to a more accurate sampling of this surface with ARMA than with a phased array, especially if conformal pixels are used (Figure 5) to maintain aerodynamics.

**Figure 29:**
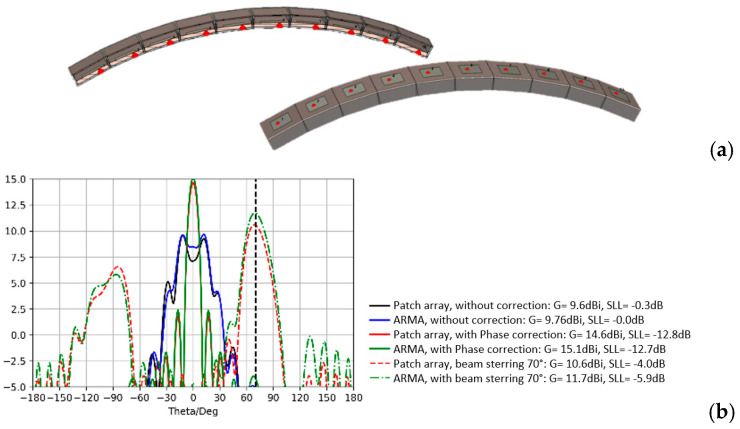
(**a**) Conformal ARMA and AESA. (**b**) Radiation pattern comparison for steering in the 60° direction.

For example, on an ARMA and AESA conformed on a cylindrical surface with a bending angle of 60° (Figure 29a), a big difference appears between the radiations at 70° (Figure 29b) through a level of grating lobes much higher with the phased arrays.

### 3.5. Multifunctionality

ARMA offers many more possibilities than arrays for building multifunctional antennas, thanks to the wide variety of pixel and matrix shapes, bandwidth, adaptation with many excitation probes, even simultaneously. Two example are given.

#### 3.5.1. Band Sharing

**Figure 30:**
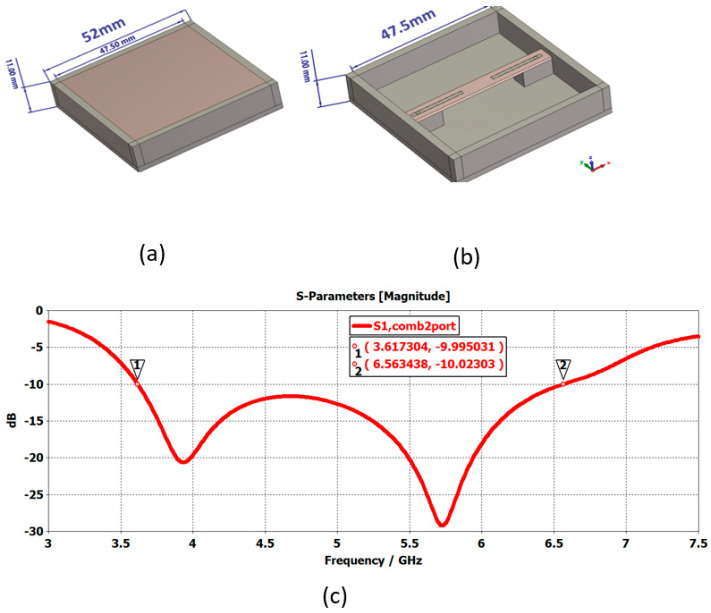
(**a**) Wideband pixel, (**b**) pixel without FSS, and (**c**) reflection coefficient of the pixel. Markers 1 and 2 correspond to the bandwidth limits.

In the standard C band communications [14], the TX band runs from 5.850 GHz to 6.425 GHz, and the RX band from 3.625 GHz to 4.200 GHz. If, in the matrix, we want to use a single pixel for both functions, its band must run from 3.6 GHz to 6.6 GHz, i.e., 58% of the band (Figure 30).

#### 3.5.2. Generation of Orthogonal Polarization (Dual Polarization)

**Figure 31:**
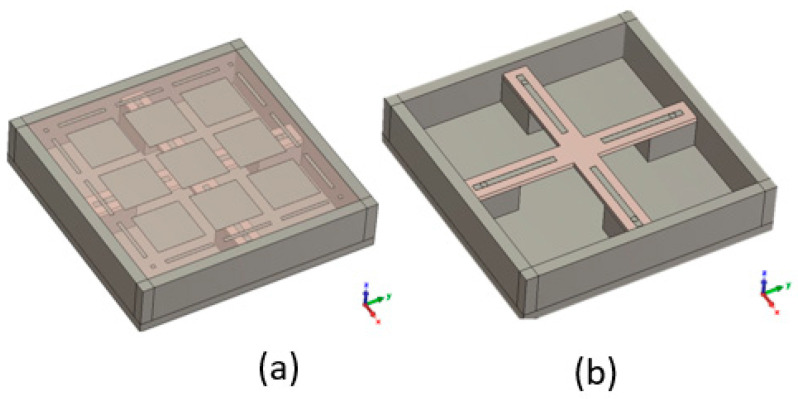
Pixel with two dipoles each formed of two monopoles: (**a**) with and (**b**) without the PRS.

A dual-polarized pixel can be obtained with an excitation in the xoz plane and another in the yoz plane. For example, by using two dipoles, each formed two monopoles excited in phase opposition (Figure 31).

**Figure 32:**
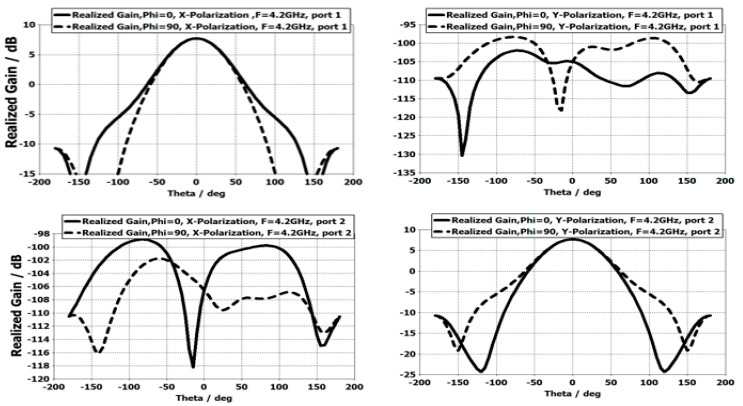
Co- and cross-polar radiation patterns of the pixel for each excitation dipole: port 1 and port 2.

If the probes are perfect, the pixel gain, if considered as a 1-element ARMA, shows highly decoupled patterns in the two polarizations, one along the x axis and the other along the y axis (Figure 32).

The operating bands on the two ports are practically identical: S11(f) ≈ S22(f) (Figure 32), and the decoupling S12(f) is excellent. However, be careful; this result will be strongly degraded by real excitation circuits and manufacturing defaults that are far from perfect.

#### 3.5.3. Shared Aperture Antennas

**Figure 33:**
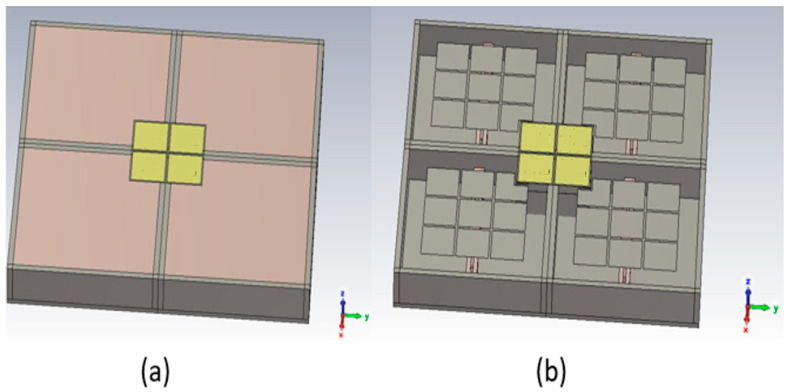
Shared Aperture Antenna (SAA) formed in two ARMAs, (**a**) 3D view of the complete antenna, and (**b**) view without the S-band substrate FSS.

When the functions to be performed involve very different frequency ranges, it is possible to use antennas placed one inside the other. They share the same radiating surface and must not interfere with each other: these are Shared Aperture Antennas (SAAs) [15,16]. It can be performed with ARMA. As an example, let us consider an S-band four-pixel square ARMA; each pixel is fed by a dipole made up of two monopoles (Figure 29). On the same surface, an X-band four-pixel square is placed (each X-band pixel is fed by a patch) (Figure 9). The ARMA SAA is shown in Figure 33.

**Figure 34:**
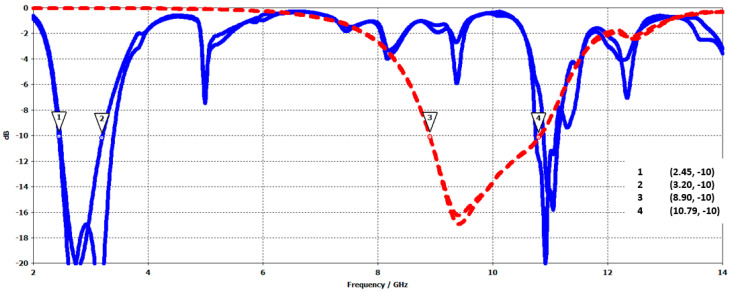
Reflection coefficient of the 8 pixels of the S-band antenna and 4 pixels of the X-band antenna, the blue curves correspond to S-band pixels and the red curves correspond to X-band pixels. (markers 1 and 2 correspond to the lower bandwidth, and markers 3 and 4 correspond to the upper bandwidth).

Figure 34 shows the reflection coefficient of the two antennas forming the SAA. It presents two operating bands. The S-band antenna, with its higher modes, partially interferes with the X-band antenna by being matched in part with its operating band, which can cause problems. Filtering can solve this issue.

**Figure 35:**
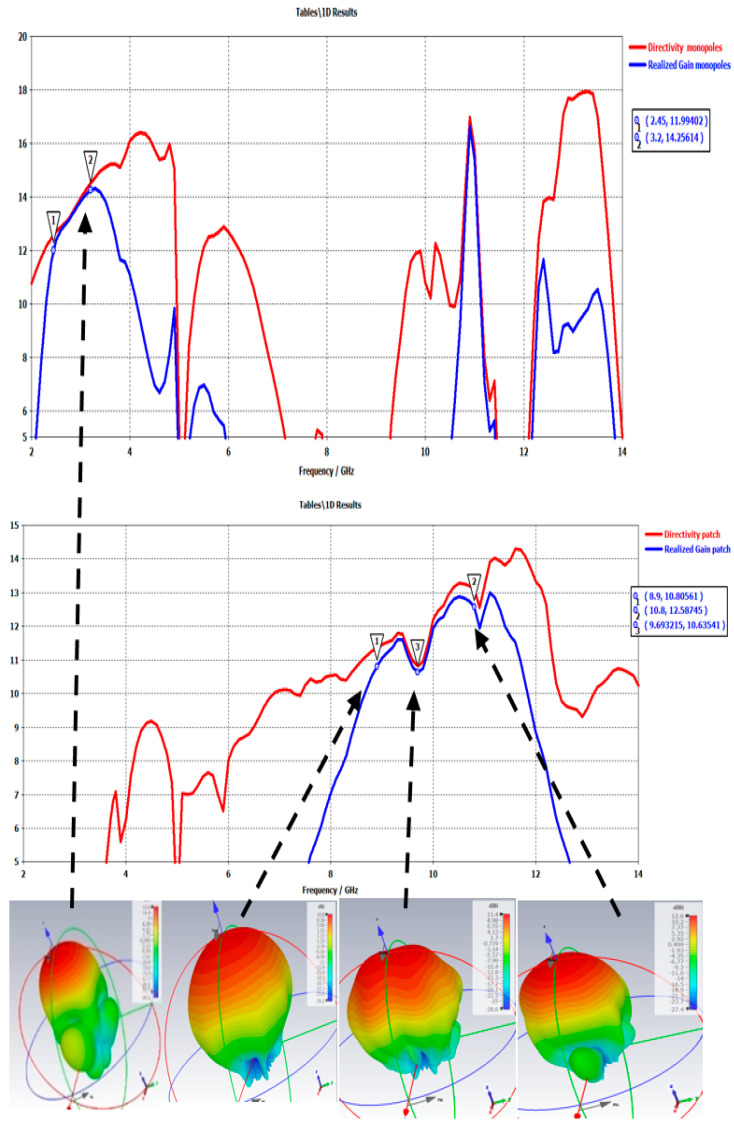
Evolution of S-band antenna and X-band antenna gains according to the frequency and maps for some frequencies.

The max gain of the S-band antenna is slightly disturbed by the presence of the X-band antenna: −0.3 dB (radiating surface reduced). Moreover, the max X-band gain has some minor ripples due to the presence of the S-band antenna (Figure 35).

## 4. Conclusions

The aim of this paper is to give a fairly comprehensive overview of the ARMA technique, both in theory and in application.

On the theoretical level, it is clearly demonstrated that ARMA is a more accurate approach than phased arrays (finer approximation of the ideal case). The applications developed all corroborate this result, but will only show their full advantage for emerging communications requiring very wide bandwidths, significant beam shapes and pointing, and integration on complex structures. The only disadvantage in relation to the arrays is in the manufacturing process, which is a little more difficult: a PRS and vertical walls have to be added.

Like arrays in their early days, the ARMA technique offers a wide range of applications, particularly in conformation and multifunctionality, whether in terrestrial or space communications, radar or EW, which are not yet exploited.

## Abbreviations

The following abbreviations are used in this manuscript:

AESA	agile electronically scanned array
ARMA	agile radiating matrix antenna
EBG	electromagnetic band gap
FSS	frequency selective surface
PRS	partially reflecting surface
